# Childhood Trauma Questionnaire (CTQ) in Brazilian Samples of Different Age Groups: Findings from Confirmatory Factor Analysis

**DOI:** 10.1371/journal.pone.0087118

**Published:** 2014-01-27

**Authors:** Rodrigo Grassi-Oliveira, Hugo Cogo-Moreira, Giovanni Abrahão Salum, Elisa Brietzke, Thiago Wendt Viola, Gisele Gus Manfro, Christian Haag Kristensen, Adriane Xavier Arteche

**Affiliations:** 1 Centre of Studies and Research in Traumatic Stress, Post-Graduate Program in Psychology, Pontifical Catholic University of the Rio Grande do Sul (PUCRS), Porto Alegre, RS, Brazil; 2 Department of Psychiatry, Universidade Federal de São Paulo, São Paulo, SP, Brazil; 3 Department of Psychiatry, Universidade Federal do Rio Grande do Sul, Porto Alegre, RS, Brazil; 4 Centre of Studies and Research in Traumatic Stress, PUCRS, Porto Alegre, RS, Brazil; University of Stellenbosch, South Africa

## Abstract

The Childhood Trauma Questionnaire (CTQ) is internationally accepted as a key tool for the assessment of childhood abuse and neglect experiences. However, there are relative few psychometric studies available and some authors have proposed two different factor solutions. We examined the dimensional structure and internal consistency of the Brazilian version of the CTQ. A total of 1,925 participants from eight different clinical and non-clinical samples including adolescents, adults and elders were considered in this study. First, we performed Confirmatory Factor Analysis to investigate the goodness of fit of the two proposed competitive factor structure models for the CTQ. We also investigated the internal consistency of all factors. Second, multi-group analyses were used to investigate measurement invariance and population heterogeneity across age groups and sex. Our findings revealed that the alternative factor structure as opposed to the original factor structure was the most appropriate model within adolescents and adults Brazilian samples. We provide further evidence for the validity and reliability of the CTQ within the Brazilian samples and report that the alternative model showed an improvement in fit indexes and may be a better alternative over the original model.

## Introduction

The Childhood Trauma Questionnaire (CTQ) is a screening tool that aims to detect experiences of childhood abuse and neglect in adults and adolescents [1], [2]. It has been shown to be the most widely used instrument to screen for emotional (EA), physical (PA) and sexual (SA) abuse and emotional (EN) and physical neglect (PN) that occurred during childhood. For instance, a Medline search carried out in September 2013 revealed that the CTQ had been used in more than 339 different studies. Moreover, the CTQ seems to be more reliable compared to other methods of assessing childhood maltreatment such as staff observations and parental reports [3].

Childhood trauma has become a hot topic of research since a variety of studies have documented its life-long impact on mental and physical health [4], [5]. The CTQ is internationally accepted as a key tool for the assessment of traumatic childhood experiences in different countries [6]–[15], including the Brazilian-Portuguese version [16]. Although the instrument is widely used and has demonstrated evidence of validity and reliability in several countries, the normative values and factor structure are still a topic of debate.

With respect to normative data, the original norms were derived from six different samples with only three of them having more than 300 subjects. These samples included mostly male adult substance abuse inpatients, adolescent psychiatric inpatients and female members of an unspecified health maintenance organization. Despite using a relatively non-representative sample, the manual establishes norms for each of these subsamples that were also used to create four severity categories [1]. However, it is yet to be known if this classification system can be applied to other samples. More importantly, the CTQ psychometric properties across different age groups, from pre-adolescence to the elderly, are still to be better investigated.

Based on its theoretical background, CTQ items should load in five different factors [1]. Nevertheless, studies that investigated the CTQ's factor structure have found mixed results, with some suggestions that its structure may vary across sample groups. In particular, the subscale PN was found to load in different factors [6], [10], [17]. Researchers suggest that this issue could be related to problems in the original CTQ construction and not a weakness of the different language versions. Furthermore, researchers argue that this problem may be due to the poor differentiation between PN and EN or because these two separate factors are conceptually intermingled in the construct of physical neglect [11]. This view was supported by two different studies in which factor analysis showed that the item number 2 (the knowledge about the existence of someone to take care and protect the participant during childhood) and number 26 (the knowledge about the existence of someone to take the participant to see a doctor if needed during childhood) loaded on the EN scale instead of the proposed PN scale [10], [11]. This finding suggests a different factor structure than that originally proposed in the manual [1]. In fact, conflicting results concerning the stability of the five-factor model have been described since Wright et al. [18] found that this model was significantly less stable for female compared to male students.

Considering the importance of the CTQ in the current literature, the relative few psychometric studies available and the evidence for the existence of two different factor solutions, the current study examined the dimensional structure and internal consistency of the Brazilian version of the CTQ [16] in clinical and community samples of three different age groups: adolescents, adults and elders. Two competing structures were examined: (a) the original five-factor solution suggested by Bernstein and Fink [1] and (b) the alternative five-factor solution with items number 2 and 26 loaded on the EN instead of on the PN scale first proposed by Gerdner and Allgulander [10]. To investigate a common latent trait, both structures were tested as loaded on a second order factor.

## Methods

### Sample

The CTQ scores of 1925 participants from eight different samples were included; 54.5% were adolescents (n = 1050), 26.4% were adults (n = 509) and 19% were elders (n = 366), with 64.56% women. These samples were selected from clinical and non-clinical samples as described below. This research was approved by the Scientific Review Board of the Psychology Faculty of Pontifical Catholic University of Rio Grande do Sul (PUCRS). In addition, the Ethics Committees of PUCRS, Universidade Federal de Ciências da Saúde de Porto Alegre and Hospital de Clínicas de Porto Alegre also approved all procedures regarding each sample. All adult participants provided written informed consent and the written informed consents from the caretakers or guardians were also obtained on behalf of the minors/children participants involved in this study.

#### Clinical samples (n = 364)

(1) Patients with panic disorder who were at least 18 years old were selected from the anxiety disorder outpatient program by convenience. The participation rate was not directly measured, but can be estimated to be approximately 80% of the patients invited. Diagnosis was performed by a trained psychiatrist using the MINI International Neuro Psychiatric Interview (n = 123; 70.7% women; mean age 37.42 years, SD = 10.29). (2) Adolescents between 10 and 17 years of age with DSM-IV primary diagnoses of anxiety disorders (except for specific phobia) (K-SADS-PL) were screened in a community sample of 2754 students from the six public schools in the primary care unit from the university catchment area (n = 94; 74.5% women; mean age 13.81, SD = 1.94). The non-participation rate was 10%. (3) Women with psychiatric problems and criminal records in compulsory care were selected by convenience (n = 147; mean age 39.90 years, SD = 13.79). The participation rate was not directly measured, but was estimated to be approximately 70% of the patients invited.

#### Non-clinical samples (n = 1561)

(1) Adolescents between 10 and 17 years of age randomly were selected in a community sample of 2754 students from the six public schools near the primary care unit from the university catchment area (n = 539; 54.8% women; mean age 14.36 years, SD = 1.88). The non-participation rate (refused or not present during the research and rescue days) was 10.1%. (2) Adolescents from public schools of Porto Alegre were selected by convenience (n = 417; 61.6% women; mean age 14.61 years, SD = 1.76). The participation rate was not directly measured, but can be estimated to beapproximately 80% of the patients invited. (3) Adults who were at least 18 years old were selected by convenience within the university community (n = 116; 68.1% women; mean age 29.79 years, SD = 13.40). The participation rate was not directly measured, but can be estimated to be approximately 90% of the patients invited. (4) Adult employees from the university hospital were selected by convenience provided that they did not meet the criteria for any psychiatry disorder according to the MINI interview based on the DSM-IV diagnostic criteria. The participation rate was not directly measured, but can be estimated to be approximately 70% (n = 123; 70.7% women; mean age 37.16 years, SD = 10.11). (5) Elders (≥60 years) from a community sample were selected by convenience from a health promotion program (n = 366; 59.8% women; mean age 67.69 years, SD = 7.52). The participation rate was not directly measured, but can be estimated to be approximately 60%.

### Measures

#### The Childhood Trauma Questionnaire

The original CTQ was developed from a 70-item retrospective questionnaire for which participants were required to rate the frequency (0- never true to 5-very often true) of abuse and neglect events that took place when they “were growing up” [19]. In further studies, the length of the scale was reduced to 28 items based on exploratory and confirmatory factor analyses [2]. The therapists' ratings were used as a stringent test of the validity of retrospective reports of childhood maltreatment, and results revealed very good criterion-related validity. Convergent and discriminant validity was demonstrated using a structured trauma interview [2]. The short version of the CTQ assesses emotional abuse, physical abuse, sexual abuse, emotional neglect, and physical neglect. *Emotional abuse* refers to verbal assaults on a child's sense of worth or well being, or any humiliating, demeaning, or threatening behavior directed toward a child by an older person. *Physical abuse* refers to bodily assaults on a child by an older person that pose a risk of, or result in, injury. *Sexual abuse* refers to sexual contact or conduct between a child and an older person, including explicit coercion. *Emotional neglect* refers to the failure of caretakers to provide basic psychological and emotional needs, such as love, encouragement, belonging and support. *Physical neglect* refers to failure to provide basic physical needs including food, shelter, and safety. Each scale is presented in a 5-point Likert-type scale ranging from 5 to 25. The final scores are *classified* according to manual's cut-off scores for the severity of abuse and neglect: “none to minimal,” “low to moderate,” “moderate to severe,” and “severe to extreme”. Three additional items compose the Minimization/Denial subscale for detecting socially desirable responses or false-negative trauma reports. The total CTQ score takes into account the severity of multiple forms of abuse and neglect. The internal consistency coefficients of the original version ranged from .61 (physical neglect) to .95 (sexual abuse) [2].

The CTQ short form translation and adaptation into Brazilian Portuguese was carried out in five steps: (1) translation, (2) back translation, (3) correction and semantic adaptation, (4) content validation by professional experts (judges), and (5) a final critical assessment by the target population using a verbal rating scale. The CTQ Brazilian version proved to be easily understandable, showing good semantic validation (for a full description see [16]).

#### Data analysis strategy

First, we performed Confirmatory Factor Analysis to investigate the goodness of fit of the two proposed competitive factor structure models for the CTQ. Given the categorical nature of the items from the CTQ we used the mean- and variance-adjusted weighted least squares (WLSMV) estimator implemented in Mplus 7.1.1. Models for goodness-of-fit were assessed with the following fit indexes: chi-square, WRMR (weighted root mean square residual), CFI (comparative fit index), TLI (Tucker-Lewis Index) and RMSEA (root mean square error of approximation). To demonstrate a good fit to the data, an estimated model should have a WRMR near or below .9, an RMSEA of near or below .06, and a CFI and TLI near or above .95 [20]–[22].

Informed by the above results, multi-group analyses were then used to investigate measurement invariance and population heterogeneity across age groups; a set of steps, from least restrictive to most restrictive, was considered as suggested by Muthén & Muthén [22]: *Step 1* - Each group (adolescents, adults and elderly) was fitted separately. *Step 2* - Threshold and factor loading were freed across the groups. Scale factors, which capture across group differences in the variances of the latent response variables for observed categorical dependent variables, were fixed at one in all groups. Factor means were fixed at zero in all groups. *Step 3* - Thresholds and factor loadings were constrained to be equal across groups; scale factors were fixed at one in one group and free in the others; factor means were fixed at zero in one group and free in others.

Due to the CTQ's ordinal outcomes, measurement invariance models constrain thresholds and factor loading in tandem because the item probability curve is influenced by both parameters. Delta parameterization and the weighted least squares estimator were used.

## Results

### Dimensionality and Internal Consistency

**Figure 1:**
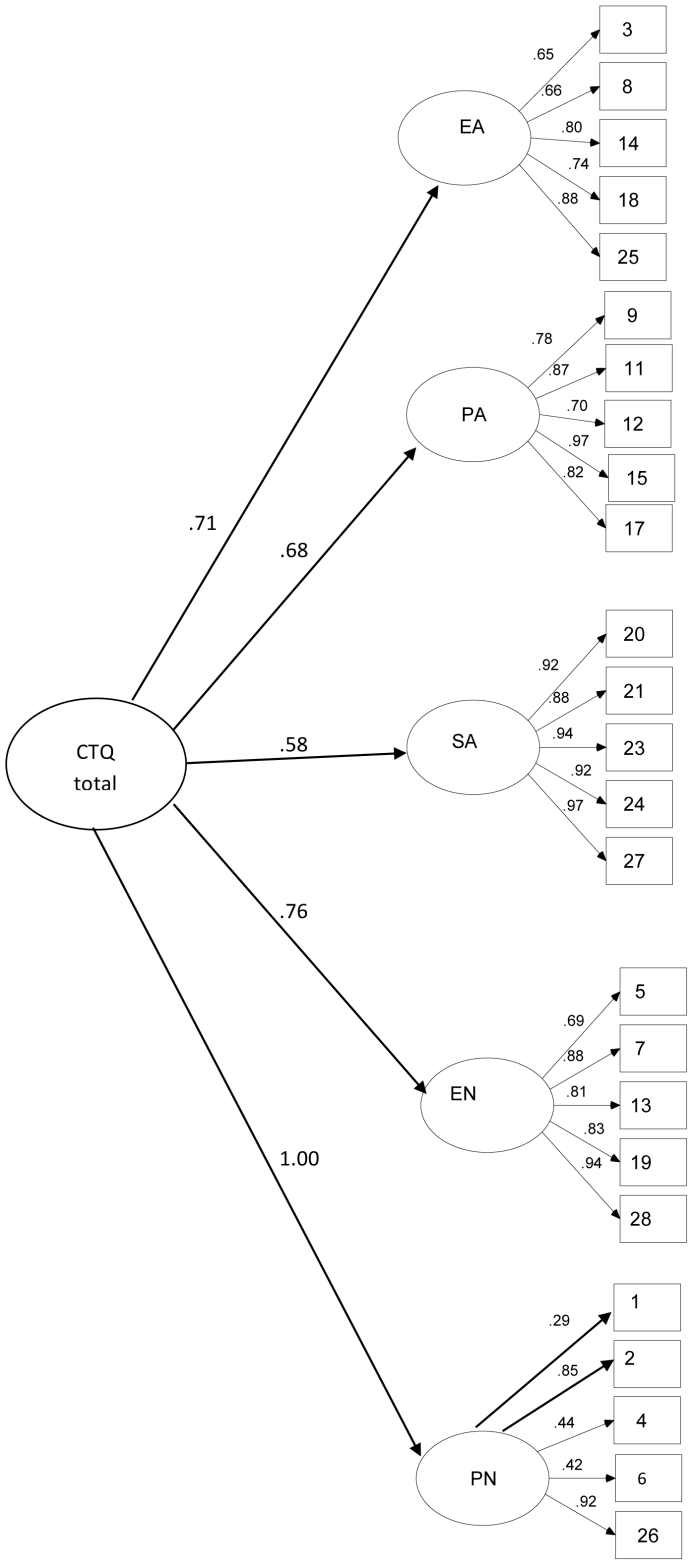
Confirmatory Factor Analysis of the Original Model in the Overall Sample. Legend: Values are Standardized Regression Weights for Items in the CTQ.

Our results revealed that the original model was not supported for our overall sample [χ^2^(270) = 5132.43, *p*<.0001; CFI = .88; TLI = .87; RMSEA = .097, confidence interval [CI] 90% = .094, .099] (Figure 1). Indeed, the original model returned a non-positive latent variable covariance matrix (ψ), indicating a negative variance/residual variance for a latent variable, a correlation greater or equal to one between two latent variable, or a linear dependency among than two latent variable; such solution, per se, is inadmissible; therefore, the model must be change.

In accordance with what was observed in the Swedish version of the CTQ [10], three items showed a relatively low loading (item 1 =  .29, item 4 = .44 and item 6 = .42) on the latent structure of factor five (PN).

**Figure 2:**
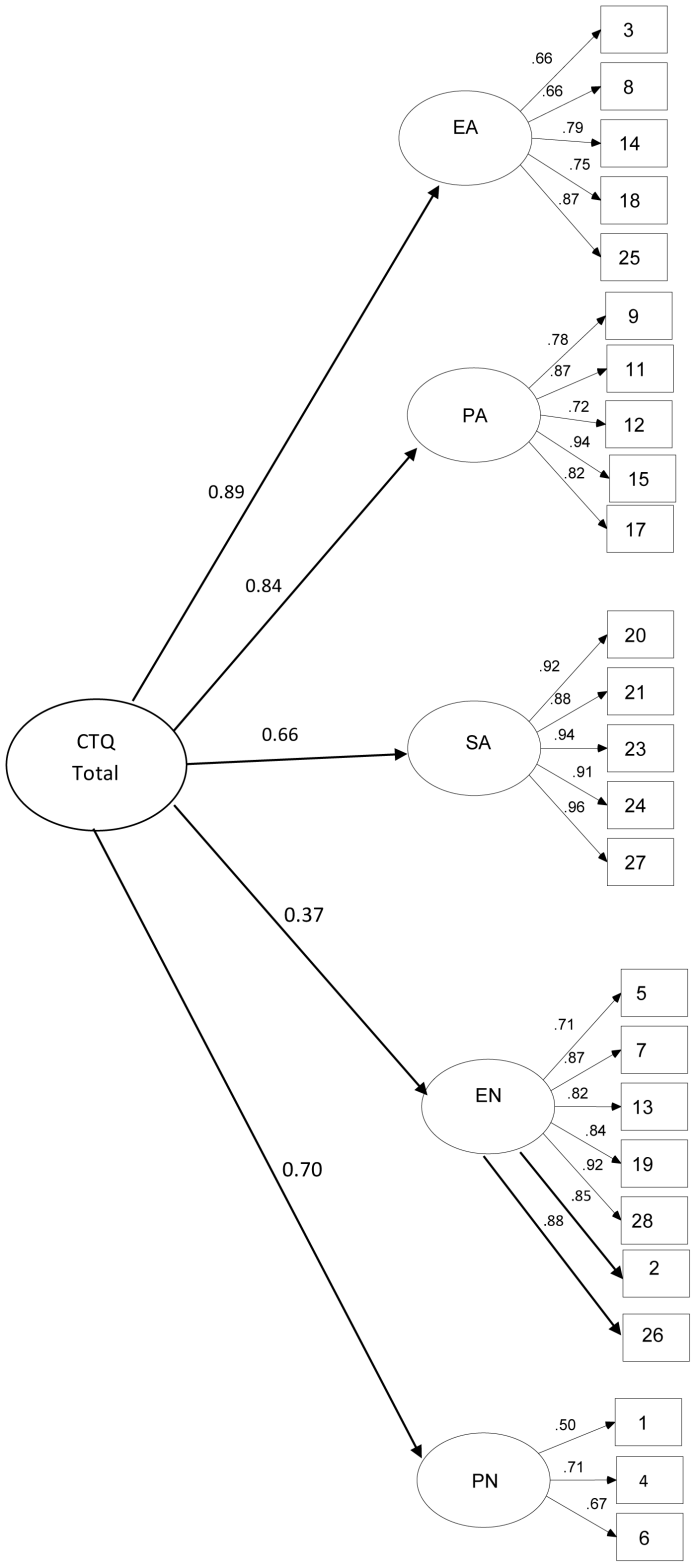
Confirmatory Factor Analysis of Alternative Five-Factor Model in Overall Sample. Legend: Values are Standardized Regression Weights for Items in the CTQ.

Therefore we did test an alternative five-factor solution and it showed an improvement in the model parameters, and all fit indexes were within the expected range (χ^2^(270) = 1174.22, *p*<.0001; CFI = .98; TLI = .98; RMSEA = .04, confidence interval [CI] 90% = .039,.044 (Figure 2).

To overcome the disadvantage of Cronbach's alpha, which has strongly been criticized in the psychometric literature [23] (e.g., it is not derived from parameters of a factor model, using the variances and covariances among the items, for major details see [24]), the scale reliabilities were estimated based on results of CFA [25], [26]. The values for the alternative model are as following: EA = 0.88, PA = 0.92, SA = 0.97, EN = 0.94, and PN = 0.66. Since the original model returned poor fit models and a non-positive latent variable covariance matrix due to correlation between PN and T factors greater than 1 (being a inadmissible solution, as consequence), we decide to no report the scale reliability for original model [1]. However to compare with the original and previous adaptations of the CTQ, Cronbach's alpha is also reported. We found that for the alternative five-factor solution Cronbach's alpha revealed acceptable levels of internal consistency (EA α = .80, PA α = .80, SA α = .90, EN α = .91). The only exception was the PN subscale (less items) that showed weak index (α = .46).The correlation coefficients among CTQ subscales are shown in Table 1.

**Table 1:** Inter-correlations among Childhood Trauma Questionnaire subscales.

	PA	SA	EN	PN	ENm	PNm
EA	.46	.30	.28	.28	.25	.30
PA	–	.26	.18	.21	.19	.28
SA		–	.17	.20	.18	.20
EN			–	.66	.97	.09
PN				–	.75	.61
ENm					–	.09
PNm						–

Note: All correlations were deemed significant at p<.01. Correlations were calculated as Pearson*r*values, except for correlations with PA and SA. Given the skewness>2.50, coefficients involving these subscales refer to Spearman's rho. Abbreviations. EA = Emotional Abuse. PA = Physical Abuse. SA = Sexual Abuse. EN = Emotional Neglect. PN = Physical Neglect. ENm = Emotional Neglect Modified. PNm = Physical Neglect Modified.

### Multi Group Analyses

**Figure 3:**
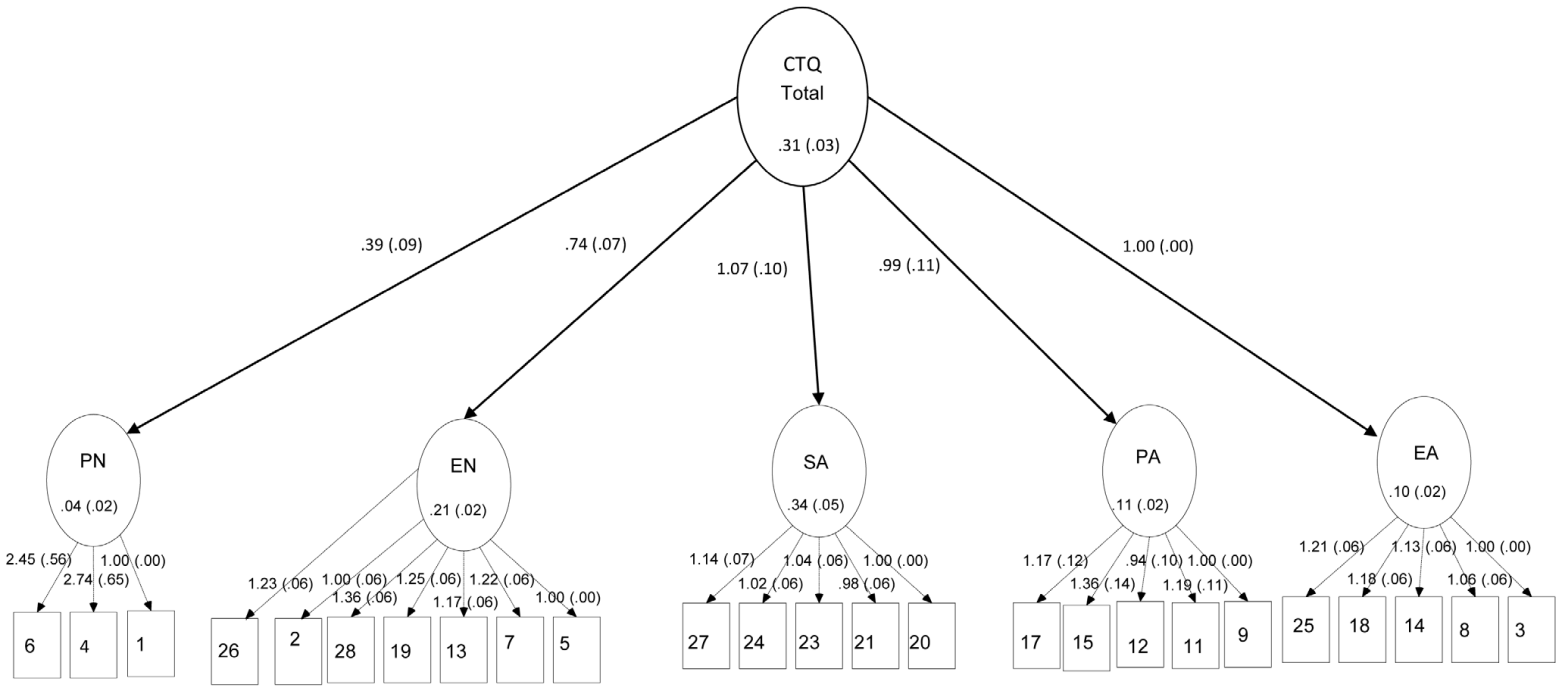
Multiple Indicator Multiple Indicator Cause (MIMIC) Revealing Acceptable Fit Indexes for Adolescents. Legend: Values are Standardized Regression Weights for Items in the CTQ.

**Figure 4:**
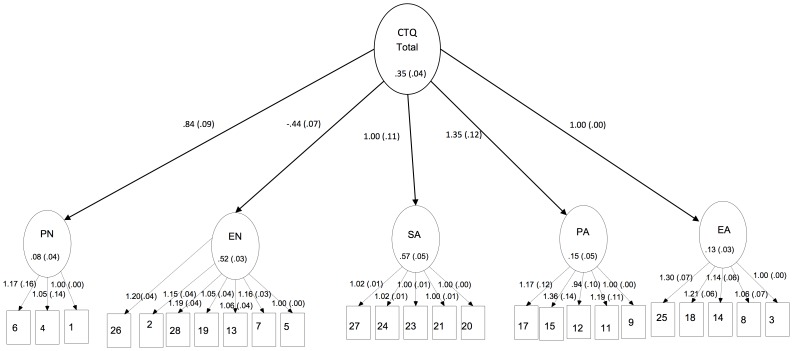
Multiple Indicator Multiple Indicator Cause (MIMIC) Revealing Acceptable Fit Indexes for Adults. Legend: Values are Standardized Regression Weights for Items in the CTQ.

Given the heterogeneity of the sample, multi-group analyses were performed to investigate the measurement invariance across multiple age groups (adolescents, adults and elderly). Considering both the CFA and the internal consistency results, our findings supported the alternative five-factor solution as the most appropriate for the Brazilian sample. Therefore, multi-group analyses are shown only in this model. Multiple Indicator Multiple Indicator Cause (MIMIC) results, separately for each group, revealed acceptable fit indexes for adolescents [CFI = .954, TLI = .949, RMSEA = .04 [.036–.043]] (Figure 3) and adults [CFI = .991, TLI = .99, RMSEA = .045 [.039–.05]] (Figure 4). However, the elderly group had a residual covariance matrix (theta), which was not definitely positive. Due to this inadmissible solution, we did not proceed with the elderly group in the following more restrictive models. Intercepts, factor loadings and factor means of the adolescent and adult groups were fixed/freed as stated in Step 2, resulting in poor fit indexes [CFI = .939, TLI = .935, RMSEA = .074[.072–.077]]. We did not execute Step 3 because in Step 2, which is less restrictive, it non-invariance was observed.

In order to test the configural invariance of the second-order alternative model considering sex, it was obtained model fits for each age group. The female adolescent model had the following fit model indexes: χ^2^(270) = 541.701, p<0.001; RMSEA = 0.040 (90%IC = 0.035–0.045); CFI = 0.961; TLI = 0.957; WRMR = 1.178. Regarding the male adolescent model, the residual covariance matrix (theta) was not positive definite. It could indicate a negative variance/residual variance for an observed variable, a correlation greater or equal to one between two observed variables, or a linear dependency between more than two observed variables. After checking the results, it was identified a problem involving item 27 (participant has to answer if he/she believes he/she was sexually abused); therefore this solution is inadmissible, being necessary to re-specify the model. After the exclusion of such an item, the fit models are marginally good: χ^2^(247) = 541.701, p<0.001; RMSEA = 0.040 (90%IC = 0.033–0.047); CFI = 0.946; TLI = 0.940; WRMR = 1.028. The female adult model had the following fit model indexes: χ^2^(270) = 493.344, p<0.001; RMSEA = 0.047(90%IC = 0.040–0.053); CFI = 0.990; TLI = 0.988; WRMR = 1.142. Considering the adult male subsample, an amount of zero cells in the bivariate table appeared together with the residual covariance matrix, indicating perfect correlation between several items (mainly related to sexual abuse). Therefore, more than to exclude items, a deeper re-specification of the model regarding such sample need to be done. Since it is necessary to solve the amount of zero cells in the bivariate table, we did not report the fit model indexes. Lastly, taking into account the female elderly model, good indexes were observed: χ^2^(270) = 333.450, p = 0.005; RMSEA = 0.032(90%IC = 0.018–0.043); CFI = 0.99; TLI = 0.989; WRMR = 0.803. However, the same problems of adult male model structure were observed in the elderly model: an amount of zero cells in the bivariate table appeared together with the residual covariance matrix, therefore we did not report the fit model indexes.

Overall, the CTQ Minimization/Denial scale showed that 5.1% of adolescents, 4.7% of adults and 17.2% of elderly participants minimized their reported childhood maltreatment experiences.

## Discussion

The CTQ is a 28-item retrospective self-report questionnaire with strong psychometric properties for clinical and non-clinical samples that has been used in many different countries. In this study, we investigated the dimensionality and model fit in a Brazilian sample. Our findings revealed that an alternative factor structure, as opposed to the factor structure from the original model, was the most appropriate model for Brazilian samples. Item numbers 2 and number 26 loaded on the EN scale instead of the proposed original PN subscale, in accordance with previously reported studies in other cultures [10], [11]. In addition, adolescents and adults revealed acceptable fit indexes, and the overall CTQ subscales have good internal consistencies. For each age group, the configural invariance regarding sex was demonstrated; it indicates that women presented good fit indexes within all age groups, however for men the observed indicators of the CTQ measure different constructs, especially regarding sexual abuse within adults and elders, thus further invariance tests are unwarranted (for example, weak measurement invariance, strong measurement invariance).

The major differences between the original and the alternative solutions are related to the definition of child neglect. Neglect is a difficult construct to operationalize because most definitions are based on personal perceptions of (the lack of) care. Specifically, child rearing practices are very different across cultures; however, the lack of parental care and the failure to meet children's basic needs seems to adversely impact child development across cultures and is broadly defined as neglect [27], [28]. The CTQ manual defines PN in terms of whether the respondents did not have enough to eat, if their parents' drinking interfered with their care, if they ever wore dirty clothes, and if there was someone to take them to the doctor. On the other hand, EN is defined considering whether their family made them feel special and loved and if their family was a source of strength, support, and protection [19]. Considering that a traditional list of immediate basic needs includes food, shelter and clothing [29], any failure to provide such basic physical safety and security could be considered physical neglect. Therefore, it is possible that EN can occur even when physical needs are met, and PN can exist even in families that are not particularly caring and supportive (especially in poverty contexts). However, Gerdner and Allgulander [10] suggest that items 2 and 26 refer to the lack of care, which is a concept with emotional connotations. The modified PN subscale includes item 4 (parents too drunk or high to take care of the family), which focuses primarily on the parents misuse problems rather than whether care is actually lacking, and also includes item 1 (food) and item 6 (clothing). All of these items loaded on physical neglect as expected and despite the low internal consistency of PN detected (0.66) it is similar to the original manual.

It is required that assessments of substantive hypotheses regarding group differences demonstrate structure measurement invariance, regardless of the type of comparison (from a simple between-group mean differences test to structural equation modeling) [30]. Although rarely tested, assumptions related to measurement invariance are routinely and straightforwardly testable. If they are not tested, violations of measurement equivalence assumption (i.e., across ages, gender, different socio-economic status) are as threatening to substantive interpretations as the inability to demonstrate reliability and validity [30]. We found that CTQ's latent structure is not invariant across the ages, resulting in an inadmissible solution for the elderly group due to a non-positive covariance matrix (theta). This lack of invariance might be related to: 1) a negative residual variance from an observed factor, 2) a correlation greater or equal to one between two observed variables, or 3) a linear dependency among more than two observed variables. This problem might be solved if we fix the residual variance at zero. However, this issue means that the first-order factor consists of a perfect indicator of the second-order factor – a strong assumption that is not supported by previous studies. Therefore, considering the findings of the CTQ's MIMIC analysis, it seems that there is a lack of conceptual equivalence of the underlying theoretical variable (the latent trait) for each age group. In turn, this finding raises concerns regarding the adequacy of between-age group comparisons (i.e., t test, ANOVA) on the non-equivalent measures [31], [32].

The breadth of child maltreatment issues cuts across cultural and national boundaries [33], [34]. Therefore, there is a need for cross-cultural research to addresses a number of problems among these multinational and multicultural populations [35]. In this regard, our effort was aimed to provide evidence to develop a reliable and cross-validated CTQ in Brazil. However, because we do not have an additional maltreatment measure, it was not possible to verify the concurrent validity. This study included nine different samples, comprising a total of almost 2,000 subjects, being the largest CTQ study so far and including a variety of sub-samples with a wide age range across the lifespan. Finally, we provide further evidence for the validity and reliability of the CTQ within a Brazilian sample, indicating that the alternative model provided a significant improvement in fit indexes and may be a better alternative over the original model.

However it is important to highlight that our data is slightly different from other studies using CFA that replied the original structure of the CTQ [14], [18], [36], [37]. In this regard our study included participants from clinical and non-clinical backgrounds, different age groups and different cultural background (Brazilians instead North-Americans or Europeans) in contrast with such previous studies that included more homogeneous samples (e.g. only clinical samples, same age group, etc). In addition most of the studies replied the five factor original structure using principal component analysis (PCA) in order to validate the latent structure underlying. Such heterogeneity in our sample should be considered in light of our results.

In summary, the model established in the study supports the general viability of the CTQ as an adequate retrospective measurement instrument for use for adolescents and adults, especially women. On a practical level, results from this study provide evidence that the classification of childhood neglect in terms of physical or emotional neglect should be re-considered in light of the alternative model in some cultures.
